# Digital Communication Biomarkers of Mood and Diagnosis in Borderline Personality Disorder, Bipolar Disorder, and Healthy Control Populations

**DOI:** 10.3389/fpsyt.2021.610457

**Published:** 2021-04-08

**Authors:** George Gillett, Niall M. McGowan, Niclas Palmius, Amy C. Bilderbeck, Guy M. Goodwin, Kate E. A. Saunders

**Affiliations:** ^1^Oxford University Clinical Academic Graduate School, John Radcliffe Hospital, The Cairns Library IT Corridor Level 3, Oxford, United Kingdom; ^2^Department of Psychiatry, Warneford Hospital, University of Oxford, Oxford, United Kingdom; ^3^Oxford Health NHS Foundation Trust, Warneford Hospital, Oxford, United Kingdom; ^4^Institute of Biomedical Engineering, University of Oxford, Oxford, United Kingdom; ^5^P1vital Products, Manor House, Howbery Business Park, Wallingford, United Kingdom

**Keywords:** bipolar disorder, borderline personality disorder, digital communications, smartphone, digital phenotyping, remote monitoring, depression, mania

## Abstract

**Background:** Remote monitoring and digital phenotyping harbor potential to aid clinical diagnosis, predict episode course and recognize early signs of mental health crises. Digital communication metrics, such as phone call and short message service (SMS) use may represent novel biomarkers of mood and diagnosis in Bipolar Disorder (BD) and Borderline Personality Disorder (BPD).

**Materials and Methods:** BD (*n* = 17), BPD (*n* = 17) and Healthy Control (HC, *n* = 21) participants used a smartphone application which monitored phone calls and SMS messaging, alongside self-reported mood. Linear mixed-effects regression models were used to assess the association between digital communications and mood symptoms, mood state, trait-impulsivity, diagnosis and the interaction effect between mood and diagnosis.

**Results:** Transdiagnostically, self-rated manic symptoms and manic state were positively associated with total and outgoing call frequency and cumulative total, incoming and outgoing call duration. Manic symptoms were also associated with total and outgoing SMS frequency. Transdiagnostic depressive symptoms were associated with increased mean incoming call duration. For the different diagnostic groups, BD was associated with increased total call frequency and BPD with increased total and outgoing SMS frequency and length compared to HC. Depression in BD, but not BPD, was associated with decreased total and outgoing call frequency, mean total and outgoing call duration and total and outgoing SMS frequency. Finally, trait-impulsivity was positively associated with total call frequency, total and outgoing SMS frequency and cumulative total and outgoing SMS length.

**Conclusion:** These results identify a general increase in phone call and SMS communications associated with self-reported manic symptoms and a diagnosis-moderated decrease in communications associated with depression in BD, but not BPD, participants. These findings may inform the development of clinical tools to aid diagnosis and remote symptom monitoring, as well as informing understanding of differential psychopathologies in BD and BPD.

## Introduction

Bipolar Disorder (BD) and Borderline Personality Disorder (BPD) are psychiatric disorders with significant morbidity and associated mortality (1, 2). Both conditions share overlapping features, meaning they can be difficult to differentiate clinically and represent a diagnostic challenge in psychiatry (3–6). This is especially salient given misdiagnosis may lead to the selection of ineffective, or even harmful, treatments (7). Alongside core features of chronic mood instability and impulsivity, both conditions feature episodic exacerbation of symptoms. Individuals with BD experience episodes of depression and mania, while individuals with BPD experience acute crises often accompanied with suicidal thoughts or actions (8). The diagnostic overlap of these presentations and the fluctuant clinical course of the two disorders means that objective markers discerning diagnosis or mood may prove clinically useful in improving the accuracy of clinical diagnosis, predicting episode course and recognizing early signs of mental health crises.

Remote monitoring is concerned with the collection of clinically relevant data in ecologically-valid settings (9). Collecting time-stamped, longitudinal data in a patient's natural environment may provide a richer phenotype of mental distress than traditional forms of clinical assessment. Digital phenotyping represents a form of remote monitoring where personal digital devices, such as smartphones or wearables, are used to collect clinically relevant data (10, 11). This data may be used to identify new behavioral digital biomarkers, leading to the identification of novel phenotypes of psychiatric disorder and mental distress (9, 12). Previous research, for instance, has identified geolocation and actigraphy variables associated with clinical features in BD (13, 14). In BPD, similar passively-recorded digital markers are likely to provide insight into psychopathology and symptoms, given that BPD patients experience alexithymia and recall bias when reflecting on symptoms between clinical encounters (15, 16).

Digital phenotyping approaches are not constrained by our current classification of mental disorders and may inform more appropriate sub-grouping for diagnosis, prediction and treatment (10). This is particularly relevant in the management of depressive symptoms, where current diagnostic classification is highly heterogeneous (17). Smartphones may represent especially useful digital phenotyping tools given their relative low cost, high-frequency use and widespread ownership among the general population (11). It has been estimated that more than 90% of the world's adult population own a mobile phone (18).

Communications may represent an especially interesting subcategory of digital phenotyping in the context of BD and BPD. Observed changes in communication, such as increased talkativeness and pressured speech, are established features and predictive factors of (hypo)mania in BD (19–21). Meanwhile, symptoms, such as anhedonia, fatigue and reduced concentration may disrupt social communication in depression (22). Digital communications may also provide an empirical approach to assess psychological theories of BPD psychopathology and therapy which focus on interpersonal dysfunction as a core feature (23–25). Smartphone communication may be associated with emotional stability and mobile phone use has been hypothesized to be implicated in interpersonal attachment style (26, 27). Therefore, digital communications data may harbor clinically relevant digital biomarkers of both mood state and diagnosis in the clinically overlapping conditions of BD and BPD.

The development of any future clinical remote-monitoring tool is likely to integrate an array of variables when making predictions about diagnosis or mood (28). Therefore, it is first necessary to identify group-level associations between communications variables, diagnosis and mood symptoms in order to guide variable selection in model development (29). Previous work has investigated objective changes in phone call and short message service (SMS) use associated with both mood state and diagnosis in BD and healthy control (HC) cohorts (28, 30–32). However, findings are conflicting. Beiwinkel et al. (31) found that the frequency of outgoing SMS messages was negatively associated with depressive symptoms but not correlated with manic symptoms, while Faurholt-Jepsen et al. (30) found that call duration, but not call or text message frequency, was associated with depressive symptoms, and call frequency, incoming call duration and outgoing SMS message frequency were associated with manic symptoms. To our knowledge, patterns of digital communication are yet to be studied in BPD cohorts. Here, we present findings from an observational study of BD, BPD, and HC cohorts using self-report mood monitoring alongside passive monitoring of digital communications. We explore associations between mood, diagnosis, trait-impulsivity, and communications variables relating to phone call and SMS messaging.

## Methods

### Participants

Data was collected as part of the Automated Monitoring of Symptom Severity (AMoSS) study, conducted between March 2014 and September 2018 (33, 34). Healthy volunteers were recruited from the community, BD and BPD participants were recruited from out-patient services or registration lists of ongoing studies. Participants were recruited for an initial 3-month study period, with an option to remain in the study for 12 months or longer. The study was observational in nature and independent of the clinical care participants received. Written informed consent was obtained from all participants. Approval was granted by the NRES Committee East of England–Norfolk (13/EE/0288) and Oxford Health NHS Foundation Trust.

Participant diagnoses were confirmed prior to study enrolment by an experienced psychiatrist (KEAS) using the Structured Clinical Interview for DSM-IV and the borderline items of the International Personality Disorder Examination (IPDE). HC status was confirmed by psychiatric assessment. Exclusion criteria for HC group were: any history of neurological disorder, head injury or major psychiatric illness, or having a first degree relative with a history of BD or BPD. Exclusion criteria for BD and BPD groups were a comorbid diagnosis of the other disorder. Due to a technical problem logging communications data, only a sub-set of the total AMoSS study population were included in this study. Our study population included a total of 55 participants; 21 HCs, 17 individuals with a diagnosis of BD and 17 individuals with a diagnosis of BPD. Demographic details by diagnostic group are reported in Table 1. The median number of weeks that participants provided digital communications and mood questionnaire data for was 21 weeks (Table 1).

**Table 1:** Population characteristics and self-rated mood by diagnostic category.

		**HC**	**BD**	**BPD**	**Total**
**Participant details**					
	Participants, *n*	21	17	17	55
	Age, mean (SD)	42.38 (11.71)	42.24 (14.24)	38 (11.39)	40.98 (12.38)
	Male gender, % *(n)*	28.57% (6)	41.18% (7)	5.88% (1)	25.45% (14)
	BIS-11, mean (SD)	54.26 (6.40)	65.41 (9.03)	65.67 (11.58)	60.80 (10.49)
	Weeks in study, median (IQR)	23 (26)	19 (19)	21 (22)	21 (24.5)
**Mood details**					
	Aggregate weeks in study, *n*	642	456	401	1,499
	Euthymic weeks, *n* (%)	540 (84.11%)	279 (61.18%)	64 (15.96%)	883 (58.91%)
	Depressed weeks, *n* (%)	99 (15.42%)	90 (19.74%)	308 (76.81%)	497 (33.16%)
	Manic weeks, *n* (%)	3 (0.47%)	70 (15.35%)	11 (2.74%)	84 (5.60%)
	Mixed weeks, *n* (%)	0 (0%)	17 (3.73%)	18 (4.49%)	35 (2.33%)
	QIDS, median (IQR)	2 (5)	4 (7)	15 (8)	5 (10)
	ASRM, median (IQR)	0 (1)	1 (4)	1 (3)	0 (2)

***p < 0.001, **p < 0.01, *p < 0.05.

### Clinical Assessments

**Figure 1:**
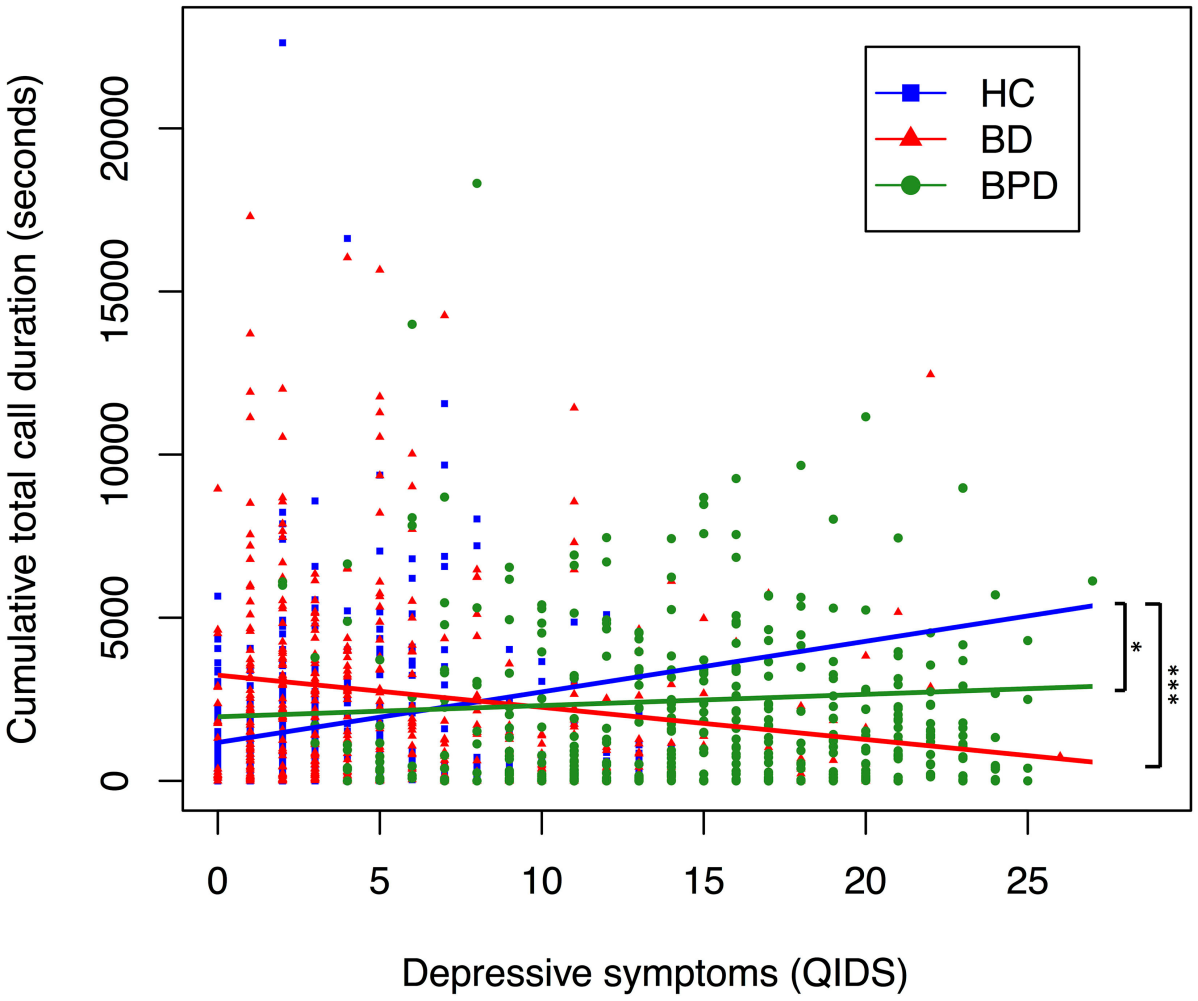
Relationship between depressive symptoms and cumulative total call duration, by diagnostic group. A scatter plot displaying the relationship between depressive symptoms and cumulative total call duration. Each point corresponds to a participants' depressive symptoms (measured by QIDS) and their cumulative total call duration (measured in seconds) in the 6 days preceding, and day of, a completed mood assessment. Color coding corresponds to diagnosis. Trendline coefficients are taken from linear mixed-effects regression models adjusted for age. Significance testing performed with HC as reference; ***p < 0.001, **p < 0.01, *p < 0.05 (HC, *n* = 642; BD, *n* = 456; BPD, *n* = 401).

Participants completed a weekly remote mood assessment using the True Colours monitoring system (35). Depressive symptoms were assessed by the Quick Inventory of Depressive Symptomatology (QIDS), manic symptoms were assessed by the Altman Self-Rating Mania Scale (ASRM) (36, 37). For mood state, thresholds of QIDS ≥11 and ASRM <6 were used to define depressive state, QIDS <11 and ASRM ≥6 were used for manic state, QIDS ≥11 and ASRM ≥6 for mixed state and QIDS <11 and ASRM <6 for euthymic state. This is in-keeping with established thresholds for moderate or severe depressive and manic episodes (36, 38). Weeks where a participant did not complete QIDS or ASRM assessments were excluded from analysis. The Barratt Impulsiveness Scale (BIS-11) was recorded upon enrolment as a measure of trait-impulsivity (39). Baseline trait-impulsivity and summary statistics for self-reported mood measures by diagnostic group are reported in Table 1. Although HC participants reported symptoms of moderate depression in a number of weeks, no HC participant reported symptoms of severe depression (QIDS ≥16) at any point in the study (Table 1; Figure 1).

### Communications Variables

Communications data were obtained from the AMoSS application that was installed on participants' smartphones at study entry. Participants without an Android device were given a smartphone and asked to use it as their primary means of communication throughout the study period. The time-stamp, length and directionality (incoming vs. outgoing) of communications (calls and SMS messages) were logged passively by the smartphone application. Weeks where a participant did not make or receive at least one phone-call or SMS were excluded from the analysis.

Selection of variables for regression analyses were guided by previous literature (28, 30). Primary communications variables were selected for their theoretical potential to directly reflect participant behavior; total and outgoing call frequency, mean total, incoming and outgoing call duration, total and outgoing SMS frequency and mean total and outgoing SMS length. Incoming call duration, but not frequency, was included due to a participant's agency to determine the length of incoming calls but not their frequency. Call and SMS frequency corresponds to the number of calls or SMS messages sent in the 6 days preceding, and day of, a completed mood assessment. Mean call duration and SMS length corresponds to the number of seconds and characters per phone-call and SMS message, respectively.

Secondary variables included cumulative duration and length, which correspond to the number of seconds or characters aggregated across all calls or text messages in the 6 days preceding, and day of, a completed mood assessment. These were selected in-line with previous methods of reporting call duration and SMS length in the literature (30) and to give a general measure of use of a communications modality (i.e., phone call or SMS messaging). Finally, variables summarizing the ratio of total and outgoing call frequency to SMS frequency and the ratio of total and outgoing call duration to SMS length were developed to standardize participants' propensity for vocal communication to their propensity for written communication, where higher values indicate a preference for vocal communication.

### Statistical Analysis

Linear mixed-effects regression models were performed with each communications variable of interest entered as a dependent variable, defined a priori. Random-effects models were used, with participant identification number entered as a random intercept. Age, diagnosis, mood state, mood symptoms, and trait-impulsivity were included as fixed effects to investigate the association between covariates and communications variables of interest. Interaction terms were inputted where relevant. Fixed effects and interaction terms are listed for each model. It was not possible to include gender as a fixed effect due to the high preponderance of female participants in our BPD sample, representative of the wider clinical population (40). Therefore, to investigate the possible effect of gender, models were replicated with gender included as a fixed effect for the BD and HC cohorts only (Supplementary Material). For mood state, euthymic state was used as a reference level in dummy coding. For diagnosis, HC was used as a reference level in dummy coding, apart from where stated otherwise. Regression analyses were performed with lmerTest (41) package in R (42), which performs *t*-tests using Satterthwaite's method for each covariate; *p*-values below 0.05 were considered statistically significant. Consistent with previous research, we report unstandardized coefficients (notated as B); which represent the amount (in frequency of calls/messages, seconds of call, or number of characters) by which the dependent communications variable changes for a change in the stated independent variable of one unit, keeping other independent variables constant (30). Where diagnosis was included as an independent variable, it was coded as 0 or 1 using dummy coding, and therefore in such cases the unstandardized coefficient represents the difference between the diagnostic groups, keeping other independent variables constant.

## Results

### Mood

#### Mood Symptoms

Across the cohort, manic symptoms were positively associated with total call frequency (B = 0.27, SE = 0.10, *p* = 0.01) and outgoing call frequency (B = 0.22, SE = 0.07, *p* < 0.01) in the transdiagnostic model (Table 2). All results remained significant when adjusted for diagnosis. Manic symptoms were also positively associated with cumulative total call duration (seconds; B = 70.91, SE = 24.33, *p* < 0.01), cumulative incoming call duration (seconds; B = 33.50, SE = 13.69, *p* = 0.02) and cumulative outgoing call duration (seconds; B = 37.70, SE = 16.35, *p* = 0.02) (Supplementary Table 1) but not mean total, incoming or outgoing call duration (Table 2).

**Table 2:** Phone call data by mood symptoms.

	**Transdiagnostic model**^****a****^			**Adjusted by diagnosis**^****b****^		
	***Coefficient***	***S.E*.**	***p-value***	***Coefficient***	***S.E*.**	***p-value***
**Total call frequency**^**c**^						
Depressive symptoms (QIDS)	−0.020	0.068	0.766	−0.049	0.071	0.488
Manic symptoms (ASRM)	0.265	0.103	0.010**	0.241	0.103	0.020*
**Outgoing call frequency**						
Depressive symptoms (QIDS)	0.011	0.046	0.808	−0.004	0.048	0.925
Manic symptoms (ASRM)	0.217	0.069	0.002**	0.203	0.070	0.004**
**Mean total call duration**						
Depressive symptoms (QIDS)	3.336	1.359	0.015*	3.485	1.514	0.022*
Manic symptoms (ASRM)	1.983	2.176	0.363	1.888	2.215	0.394
**Mean incoming call duration**						
Depressive symptoms (QIDS)	5.104	1.621	0.002**	5.714	1.891	0.003**
Manic symptoms (ASRM)	4.756	2.698	0.078	5.049	2.766	0.068
**Mean outgoing call duration**						
Depressive symptoms (QIDS)	0.106	1.622	0.948	−0.560	1.816	0.758
Manic symptoms (ASRM)	1.424	2.613	0.586	0.873	2.659	0.743

^a^Transdiagnostic model adjusted by age only. All significant results remained significant when age removed from model. ^b^Adjusted model adjusted for both age and diagnosis. ^c^Analyses were performed separately for each variable in univariate analyses and with QIDS & ASRM variables together in multivariate analyses. Results remained significant when univariate analysis performed, multivariate analyses results presented here. ***p < 0.001, **p < 0.01, *p < 0.05.

Depressive symptoms were positively associated with mean total call duration (seconds; B = 3.336, SE = 1.359, *p* = 0.015) and mean incoming call duration (seconds; B = 5.104, SE = 1.621, *p* = 0.002) and results remained significant when adjusted for diagnosis (Table 2). There was no strong evidence of a transdiagnostic association between depressive symptoms and other primary phone call variables (Table 2).

For SMS data, manic symptoms were positively associated with total SMS frequency (B = 1.62, SE = 0.40, *p* ≤ 0.01) and outgoing SMS frequency (B = 0.72, SE = 0.20, *p* < 0.01) (Table 3). All results remained significant when adjusted for diagnosis.

**Table 3:** SMS data by mood symptoms.

	**Transdiagnostic model**^****a****^			**Adjusted by diagnosis**^****b****^		
	***Coefficient***	***S.E*.**	***p-value***	***Coefficient***	***S.E*.**	***p-value***
**Total SMS frequency**^**c**^						
Depressive symptoms (QIDS)	−0.124	0.275	0.653	−0.211	0.278	0.447
Manic symptoms (ASRM)	1.618	0.402	<0.001***	1.566	0.402	<0.001***
**Outgoing SMS frequency**						
Depressive symptoms (QIDS)	−0.107	0.140	0.444	−0.151	0.141	0.283
Manic symptoms (ASRM)	0.718	0.204	<0.001***	0.691	0.204	0.001***
**Mean total SMS length**						
Depressive symptoms (QIDS)	−0.645	0.408	0.115	−0.652	0.479	0.174
Manic symptoms (ASRM)	−0.306	0.707	0.665	−0.084	0.722	0.908
**Mean outgoing SMS length**						
Depressive symptoms (QIDS)	0.362	0.373	0.333	0.319	0.410	0.437
Manic symptoms (ASRM)	0.569	0.603	0.345	0.664	0.612	0.278

^a^Transdiagnostic model adjusted by age only. All significant results remained significant when age removed from model. ^b^Adjusted model adjusted for both age and diagnosis. ^c^Analyses were performed separately for each variable in univariate analyses and with QIDS & ASRM variables together in multivariate analyses. Results remained significant when univariate analysis performed, multivariate analyses results presented here. ***p < 0.001, **p < 0.01, *p < 0.05.

There was no evidence of an association between transdiagnostic depressive symptoms and any SMS variable (Table 3, Supplementary Table 1).

#### Mood State

For phone call data, manic state was associated with increased total call frequency (B = 5.16, SE = 1.12, *p* < 0.01) and outgoing call frequency (B = 3.41, SE = 0.75, *p* < 0.01) compared to euthymia, but not mean total, incoming or outgoing call duration (Supplementary Table 2). All results remained significant when adjusted for diagnosis. Manic state was also associated with cumulative total call duration (seconds; B = 1,344.04, SE = 264.55, *p* < 0.01), cumulative incoming call duration (seconds; B = 581.58, SE = 150.39 *p* < 0.01) and cumulative outgoing call duration (seconds; B = 766.79, SE = 177.90, *p* < 0.01) (Supplementary Table 3).

Depressive state was not associated with any primary phone-call variable (frequency or mean duration) (Supplementary Table 2). However, depressive state was associated with increased cumulative incoming call duration (seconds; B = 217.23, SE = 102.74, *p* = 0.04) and a non-significant decrease in cumulative outgoing call duration (seconds; B = −164.56, SE = 122.57, *p* = 0.18) compared to euthymia (Supplementary Table 3).

For SMS data, there was no evidence of an association between depressive or manic states and SMS variables in either model (Supplementary Table 2). Mixed states were associated with increased total SMS frequency (B = 25.24, SE = 6.82, *p* < 0.01) and outgoing SMS frequency (B = 11.39, SE = 3.46, *p* < 0.01) compared to euthymia and results remained significant when adjusted for diagnosis (Supplementary Table 2).

To investigate whether the communications changes observed in mania are partially specific to vocal, rather than written, communication, we performed regression analyses for our secondary communications variables (Supplementary Table 4). There was no strong evidence of an association between manic symptoms or manic state and call frequency standardized to SMS frequency for either total or outgoing calls (Supplementary Table 4). However, increased manic symptoms were associated with both total call duration standardized to SMS length (B = 0.67, *p* = 0.03) and outgoing call duration standardized to SMS length (B = 0.49, *p* = 0.02), while manic state was associated with outgoing call duration standardized to SMS length (B = 5.94, SE = 2.57, *p* = 0.02) although did not reach significance (*p* > 0.05) for total call duration standardized to SMS length.

### Diagnosis

For phone call data, BD diagnosis was associated with increased total call frequency (B = 5.91, SE = 2.79, *p* = 0.04) compared to HC (Table 4). Results remained significant when adjusted for mood symptoms, but not mood state (Table 4, Supplementary Table 5).

**Table 4:** Phone call data by diagnosis (adjusted by mood symptoms).

	**Unadjusted**^****a****^			**Adjusted by mood symptoms**^****b****^		
	***Coefficient***	***S.E*.**	***p-value***	***Coefficient***	***S.E*.**	***p-value***
**Total call frequency**						
BD vs. HC	5.912	2.791	0.040*	5.678	2.787	0.047*
BPD vs. HC	3.561	2.835	0.216	3.962	2.945	0.184
**Outgoing call frequency**						
BD vs. HC	3.624	1.866	0.059	3.251	1.858	0.087
BPD vs. HC	2.185	1.896	0.256	2.054	1.964	0.300
**Mean total call duration**						
BD vs. HC	42.013	39.490	0.294	22.273	41.055	0.590
BPD vs. HC	37.596	40.345	0.357	−8.183	45.381	0.858
**Mean incoming call duration**						
BD vs. HC	20.257	40.202	0.618	−15.451	42.412	0.718
BPD vs. HC	46.534	41.316	0.268	−28.714	48.506	0.556
**Mean outgoing call duration**						
BD vs. HC	50.870	45.697	0.273	51.449	47.171	0.282
BPD vs. HC	38.332	46.948	0.419	44.658	52.793	0.401

^a^Unadjusted model is adjusted by age only. All significant results remained significant when age removed from model. ^b^Adjusted model adjusted for both age and mood symptoms (QIDS & ASRM). ***p < 0.001, **p < 0.01, *p < 0.05.

For SMS data, BPD diagnosis was associated with increased total SMS frequency (B = 54.52, SE = 23.79, *p* = 0.03) and outgoing SMS frequency (B = 29.05, SE = 12.32, *p* = 0.02), compared to HC (Table 5). BPD diagnosis was also associated with cumulative total SMS length (characters; B = 4,931.97, SE = 2,129.68, *p* = 0.03) and cumulative outgoing SMS length (characters; B = 2,927.61, SE = 1,384.30, *p* = 0.04) compared to HC (Supplementary Table 6), but not mean total or mean outgoing SMS length (Table 5). Results remained significant when adjusted for mood symptoms or mood state (Supplementary Table 6). BD diagnosis was associated with decreased mean total SMS length (B = −21.300, SE = 9.306, *p* = 0.027), but lost significance when adjusted for mood symptoms or state (Table 5, Supplementary Table 5).

**Table 5:** SMS data by diagnosis (adjusted by mood symptoms).

	**Unadjusted**^****a****^			**Adjusted by mood symptoms**^****b****^		
	***Coefficient***	***S.E*.**	***p-value***	***Coefficient***	***S.E*.**	***p-value***
**Total SMS frequency**						
BD vs. HC	36.311	23.501	0.129	34.506	23.354	0.146
BPD vs. HC	54.522	23.786	0.026*	55.645	23.841	0.023*
**Outgoing SMS frequency**						
BD vs. HC	18.164	12.176	0.142	17.661	12.172	0.153
BPD vs. HC	29.045	12.323	0.022*	30.265	12.419	0.018*
**Mean total SMS length**						
BD vs. HC	−21.300	9.306	0.027*	−18.306	9.799	0.067
BPD vs. HC	−8.674	9.541	0.368	−0.404	11.433	0.972
**Mean outgoing SMS length**						
BD vs. HC	−14.918	10.937	0.179	−17.776	11.269	0.121
BPD vs. HC	6.619	11.162	0.556	2.113	12.395	0.865

^a^Unadjusted model is adjusted by age only. All significant results remained significant when age removed from model. ^b^Adjusted model adjusted for both age and mood symptoms (QIDS & ASRM). ***p < 0.001, **p < 0.01, *p < 0.05.

All significant associations between BD or BPD diagnosis and communications variables were attenuated when adjusted for trait-impulsivity (Supplementary Table 7). In separate analyses, transdiagnostic trait-impulsivity adjusted for age was associated with all variables previously identified to be associated with BD or BPD diagnosis; increased total call frequency (B = 0.20, SE = 0.10, *p* = 0.05), total SMS frequency (B = 2.72, SE = 0.87, *p* < 0.01), outgoing SMS frequency (B = 1.36, SE = 0.45, *p* < 0.01), cumulative total SMS length (characters; B = 208.04, SE = 79.67, *p* = 0.01) and cumulative outgoing SMS length (characters; B = 106.20, SE = 52.76, *p* = 0.05) (Supplementary Table 8).

### Interaction: Mood and Diagnosis

**Figure 2:**
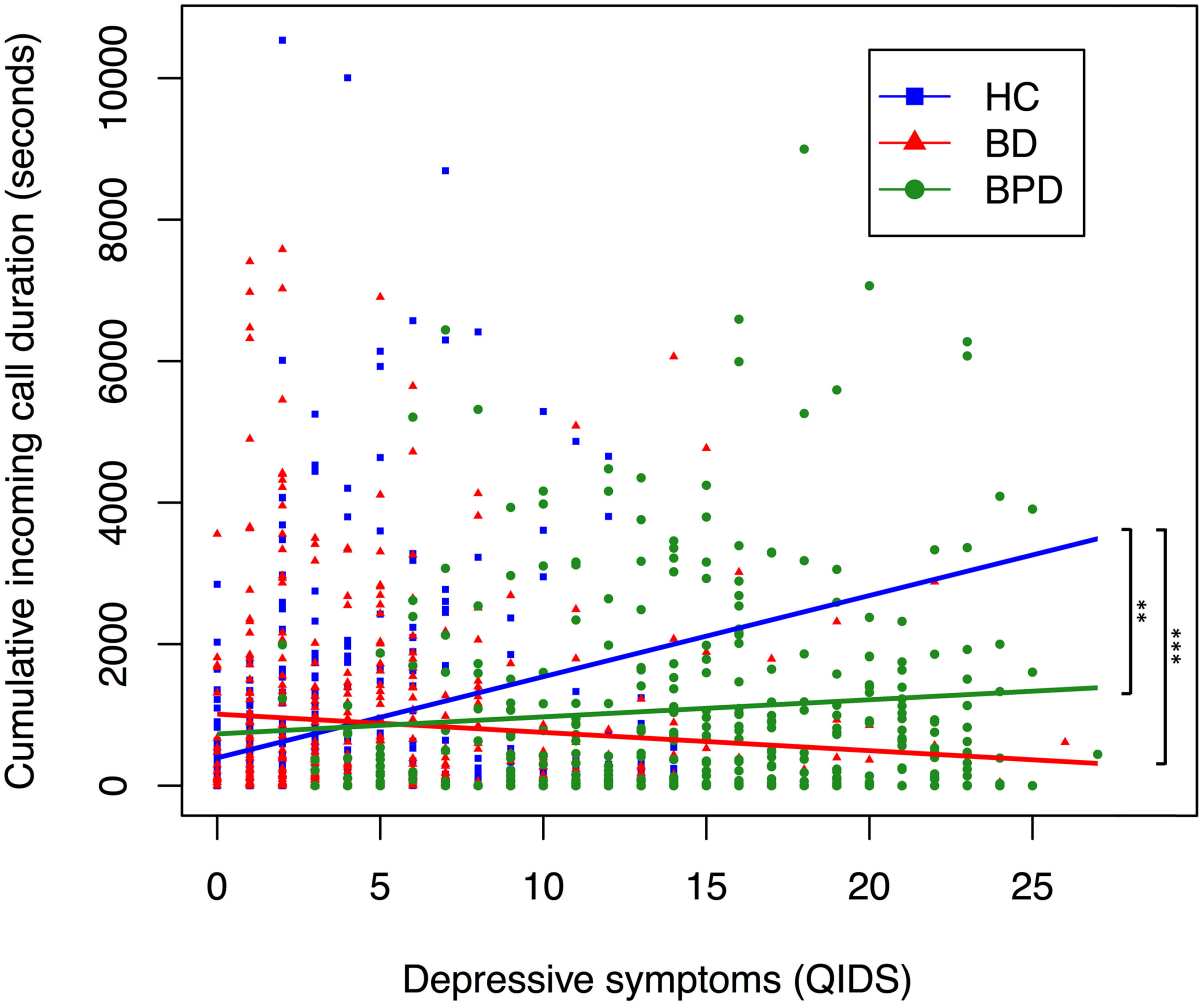
Relationship between depressive symptoms and cumulative incoming call duration, by diagnostic group. A scatter plot displaying the relationship between depressive symptoms and cumulative total call duration. Each point corresponds to a participants' depressive symptoms (measured by QIDS) and their cumulative incoming call duration (measured in seconds) in the 6 days preceding, and day of, a completed mood assessment. Color coding corresponds to diagnosis. Trendline coefficients are taken from linear mixed-effects regression models adjusted for age. Significance testing performed with HC as reference; ***p < 0.001, **p < 0.01, * *p* < 0.05 (HC, *n* = 642; BD, *n* = 456; BPD, *n* = 401).

**Figure 3:**
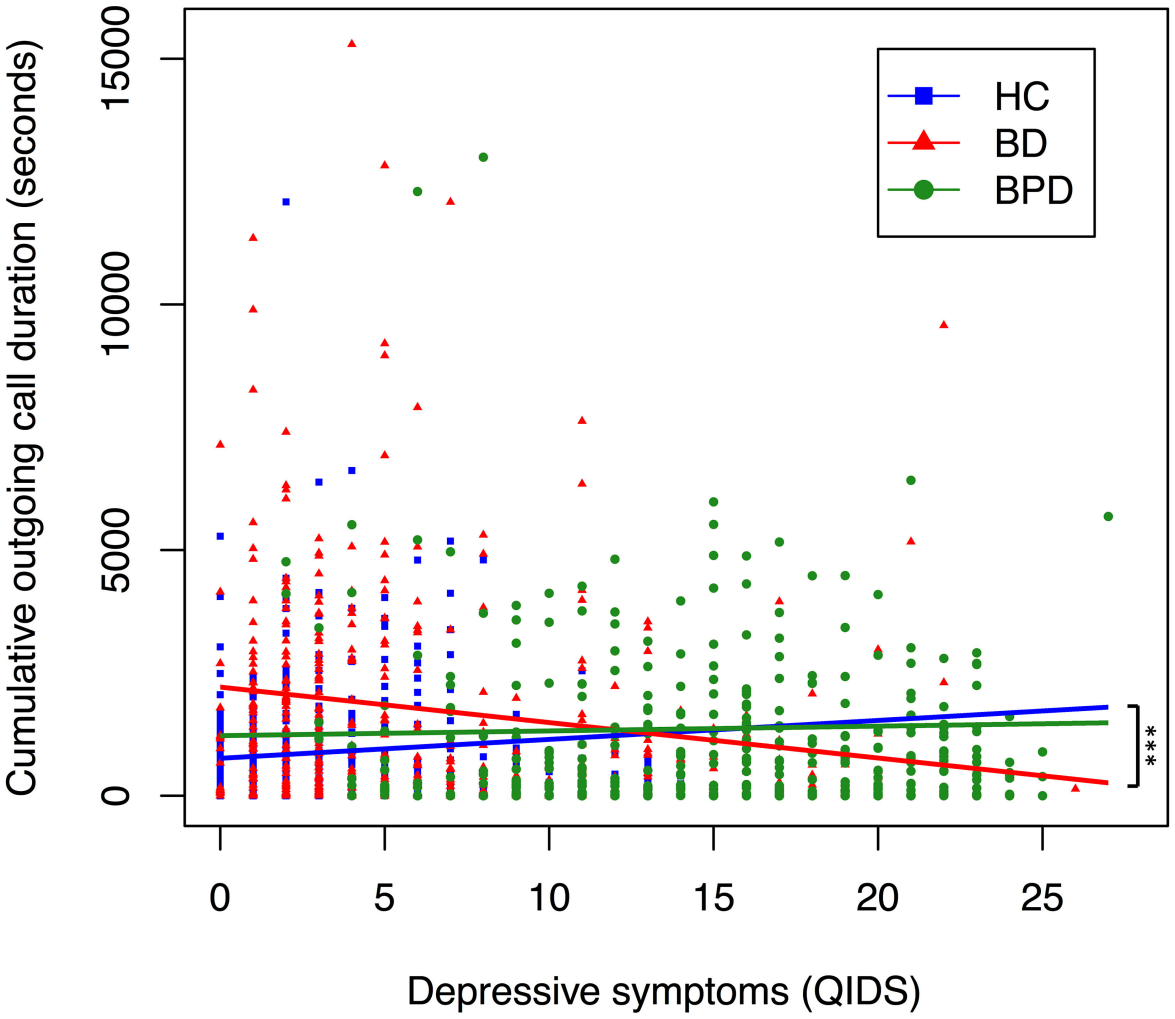
Relationship between depressive symptoms and cumulative outgoing call duration, by diagnostic group. A scatter plot displaying the relationship between depressive symptoms and cumulative total call duration. Each point corresponds to a participants' depressive symptoms (measured by QIDS) and their cumulative outgoing call duration (measured in seconds) in the 6 days preceding, and day of, a completed mood assessment. Color coding corresponds to diagnosis. Trendline coefficients are taken from linear mixed-effects regression models adjusted for age. Significance testing performed with HC as reference; ***p < 0.001, **p < 0.01, *p < 0.05. (HC, *n* = 642; BD, *n* = 456; BPD, *n* = 401).

To assess whether diagnosis moderates the effect between mood and digital communications variables, we performed regression analyses for the interaction between diagnosis and depressive state (Tables 6, 7, Supplementary Table 9). For phone call data, interaction between BD and depression was associated with decreased mean total call duration (seconds; B = −134.029, SE = 43.577, *p* = 0.002), mean incoming call duration (seconds; B = −126.671, SE = 53.030, *p* = 0.017), mean outgoing call duration (seconds; B = −126.342, SE = 53.620, *p* = 0.019), cumulative total call duration (seconds; B = −1598.62, SE = 464.92, *p* < 0.01), cumulative incoming call duration (seconds; B: −702.30, SE = 264.36, *p* = 0.01) and cumulative outgoing call duration (seconds; B = −875.95, SE = 320.467, *p* = 0.01) when HC was used as the reference dummy variable (Table 6, Supplementary Table 9). The interaction between BPD and depression was not significantly associated (*p* > 0.05) with any phone call variable other than mean incoming call duration (seconds; B = −106.436, SE = 49.903, *p* = 0.033) (Table 6). When BPD was used as the reference dummy variable, the interaction between BD and depression was associated with decreased total call frequency (B = −7.19, SE = 1.99, *p* < 0.01), outgoing call frequency (B = −3.12, SE = 1.36, *p* = 0.02), cumulative total call duration (seconds; B = −1,658.13, SE = 461.22, *p* < 0.01), cumulative incoming call duration (seconds; B = −594.73, SE = 261.93, *p* = 0.02) and cumulative outgoing call duration (seconds; B = −1,038.23, SE = 317.86, *p* < 0.01) (Table 6, Supplementary Table 9). Together these results suggest diagnosis may moderate the association between depression and phone call communications. Interaction trends between diagnostic groups and depressive symptoms for cumulative total, incoming and outgoing call duration are summarized in Figures 1–3, all other communications variables are summarized in Supplementary Figures 1,2.

**Table 6:** Phone call data by diagnosis & mood state interaction effects.

	**Reference: HC group**^****a****^			**Reference: BPD group**^****b****^		
	***Coefficient***	***S.E*.**	***p-value***	***Coefficient***	***S.E*.**	***p-value***
**Total call frequency**						
Depression	0.020	1.303	0.988	3.580	1.275	0.005**
BD vs. HC	6.941	2.715	0.014*	–	–	–
BPD vs. HC	0.972	2.910	0.740	–	–	–
BD vs. BPD	–	–	–	5.969	3.071	0.056
BD × Depression	−3.625	2.013	0.072	−7.185	1.994	<0.001***
BPD × Depression	3.560	1.823	0.051	–	–	–
**Outgoing call frequency**						
Depression	−0.453	0.888	0.610	1.593	0.868	0.067
BD vs. HC	4.063	1.750	0.025*	–	–	–
BPD vs. HC	0.856	1.886	0.652	–	–	–
BD vs. BPD	–	–	–	3.207	1.993	0.113
BD × Depression	−1.076	1.368	0.432	−3.123	1.355	0.021*
BPD × Depression	2.047	1.242	0.100	–	–	–
**Mean total call duration**						
Depression	83.598	28.283	0.003**	18.205	28.628	0.525
BD vs. HC	62.671	43.466	0.157	–	–	–
BPD vs. HC	22.739	48.917	0.644	–	–	–
BD vs. BPD	–	–	–	39.932	51.836	0.444
BD × Depression	−134.029	43.577	0.002**	−68.636	43.784	0.117
BPD × Depression	−65.392	40.241	0.104	–	–	–
**Mean incoming call duration**						
Depression	86.272	35.785	0.016*	−20.164	34.789	0.562
BD vs. HC	27.733	44.997	0.542	–	–	–
BPD vs. HC	58.665	52.537	0.268	–	–	–
BD vs. BPD	–	–	–	−30.932	55.517	0.579
BD × Depression	−126.671	53.030	0.017*	−20.235	52.331	0.699
BPD × Depression	−106.436	49.903	0.033*	–	–	–
**Mean outgoing call duration**						
Depression	28.441	35.598	0.424	6.576	36.260	0.856
BD vs. HC	93.066	50.274	0.071	–	–	–
BPD vs. HC	34.878	57.968	0.549	–	–	–
BD vs. BPD	–	–	–	58.189	61.373	0.346
BD × Depression	−126.342	53.620	0.019*	−104.477	54.038	0.053
BPD × Depression	−21.865	50.811	0.667	–	–	–

Data limited to depression & euthymia weeks (n = 1,380) to avoid rank deficiency. All analyses are adjusted for age. All significant results remained significant when age removed from model. ^a^In dummy coding, HC group used as reference level, therefore diagnosis x depression represents the moderation effect compared to reference (HC). ^b^In dummy coding, BPD group used as reference level, therefore diagnosis × depression represents the moderation effect compared to reference (BPD). ***p < 0.001, **p < 0.01, *p < 0.05.

**Table 7:** SMS data by diagnosis & mood state interaction effects.

	**Reference: HC group**^****a****^			**Reference: BPD group**^****b****^		
	***Coefficient***	***S.E*.**	***p-value***	***Coefficient***	***S.E*.**	***p-value***
**Total SMS frequency**						
Depression	0.646	4.562	0.887	2.122	4.495	0.637
BD vs. HC	41.816	30.723	0.180	–	–	–
BPD vs. HC	64.413	31.615	0.047*	–	–	–
BD vs. BPD	–	–	–	−22.597	32.840	0.495
BD × Depression	−28.781	7.180	<0.001***	−30.257	7.138	<0.001***
BPD × Depression	1.476	6.405	0.818	–	–	–
**Outgoing SMS frequency**						
Depression	−0.027	2.330	0.991	−0.101	2.296	0.965
BD vs. HC	20.689	15.558	0.190	–	–	–
BPD vs. HC	35.718	16.010	0.030*	–	–	–
BD vs. BPD	–	–	–	−15.029	16.631	0.371
BD × Depression	−12.424	3.667	0.001***	−12.350	3.646	0.001***
BPD × Depression	−0.074	3.271	0.982	–	–	–
**Mean total SMS length**						
Depression	3.506	9.930	0.724	8.771	9.553	0.359
BD vs. HC	−16.413	10.182	0.113	–	–	–
BPD vs. HC	−16.749	12.527	0.184	–	–	–
BD vs. BPD	–	–	–	0.336	13.395	0.980
BD × Depression	−18.988	14.442	0.189	−24.252	14.174	0.087
BPD × Depression	5.265	13.776	0.702	–	–	–
**Mean outgoing SMS length**						
Depression	−3.998	9.222	0.665	5.246	7.198	0.466
BD vs. HC	−13.666	11.384	0.236	–	–	–
BPD vs. HC	0.912	12.676	0.943	–	–	–
BD vs. BPD	–	–	–	−14.578	13.345	0.278
BD × Depression	−3.550	12.711	0.780	−12.794	11.322	0.259
BPD × Depression	9.244	11.697	0.430	–	–	–

Data limited to depression & euthymia weeks (n = 1,380) to avoid rank deficiency. All analyses are adjusted for age. All significant results remained significant when age removed from model. ^a^In dummy coding, HC group used as reference level, therefore diagnosis x depression represents the moderation effect compared to reference (HC). ^b^In dummy coding, BPD group used as reference level, therefore diagnosis × depression represents the moderation effect compared to reference (BPD). ***p < 0.001, **p < 0.01, *p < 0.05.

For SMS data, the interaction between BD and depression was associated with decreased total SMS frequency (B = −28.78, SE = 7.18, *p* < 0.01), outgoing SMS frequency (B = −12.42, SE = 3.67, *p* < 0.01) and cumulative total SMS length (characters; B = −1,463.73, SE = 632.69, *p* = 0.02) when HC was used as the reference dummy variable (Table 7, Supplementary Table 9). The interaction between BPD and depression was not significantly associated (*p* > 0.05) with any SMS variable (Table 7). When BPD was used as the reference dummy variable, the interaction between BD and depression was associated with decreased total SMS frequency (B = −30.26, SE = 7.14, *p* < 0.01), outgoing SMS frequency (B = −12.35, SE = 3.65, *p* < 0.01) and cumulative total SMS length (characters; B = −1,749.79, SE = 628.92, *p* = 0.01), suggesting diagnosis may also moderate the association between depression and SMS communications (Table 7, Supplementary Table 9).

## Discussion

Communications variables, incorporating phone call and SMS messaging, may represent digital biomarkers of mood symptoms, mood state and diagnosis in BD, BPD, and HC populations.

Specifically, we identified a positive association between both manic symptoms and manic state with total and outgoing phone call frequency and cumulative phone call duration, and a positive association between manic symptoms and increased total and outgoing SMS frequency. These results may reflect increased talkativeness and pressured speech, which are core features of ICD-10 and DSM-5 classification systems but are not currently operationally defined.

Furthermore, manic symptoms were associated with both increased total call duration and outgoing call duration standardized to SMS length and manic state was associated with increased outgoing call duration standardized to SMS length. This finding is novel and may refine the clinical phenotype of mania by suggesting that pressured speech and talkativeness may be objectively conceptualized as lengthening of oral relative to written communication. These results may also guide future attempts to better understand the psychopathological and neurobiological basis of the increased drive to communicate observed in manic episodes. Writing SMS messages may require a more reflective capacity than oral conversation, and be less achievable to participants during a manic episode. Alternatively, it is possible that SMS communication is simply less immediately rewarding than oral communication. Known deficits in mentalization (the ability to understand other people's mental and emotional states) associated with manic episodes may also be relevant (43). Oral communication may decrease the amount of mentalization required, by providing immediate feedback, prosodic cues and potentially less ambiguous content, compared to written messaging.

Our findings are in-agreement with previous reports that manic symptoms, measured using the Young Mania Rating Scale, are associated with increased phone call and SMS communications in a separate cohort (30). We also identified a tentative association between mixed features and increased total and outgoing SMS frequency. Adjusting for diagnosis did not affect the relationship between manic symptoms and phone use.

For depression, across the whole sample, mean incoming call duration was correlated with depressive symptoms. It is plausible that this reflects increased concern from friends and family. Alternatively, increased incoming call duration may reflect features of the depressed clinical phenotype, such as psychomotor retardation and answer latency (44, 45). The latter explanation seems less likely since depressive state was weakly associated with non-significant reductions in mean outgoing call duration, although it is possible that incoming calls present greater cognitive challenges compared to outgoing calls, exacerbating the effect of psychomotor retardation.

For depressed mood there were important effects of diagnosis. BD exhibited decreased phone call and SMS communications when depressed. This might be expected from the behavioral impact of low mood via anhedonia, fatigue, reduced concentration and motor slowing (46). In contrast, for BPD, depression was not strongly associated with any communications variable other than a reduction in mean incoming call duration.

These results add to an as yet inconsistent picture of digital communications in depressive states. While a pilot study identified decreased communications in depression, other work has identified increased phone call and SMS use in depressive states in the context of BD (28, 30, 31).

The apparent difference between the impact of depression in BD and BPD is of great interest. First, it suggests the practical possibility of a diagnostic biomarker, which would be welcome given the common clinical uncertainty in distinguishing the cause of mood instability (6). Second, while in BPD, distress is expressed in terms of depressive symptoms, they are notably more persistent than in BD (Table 1). The absence of decreased communications when depressed may be in-keeping with the clinical phenotype of BPD, where self-reported mental distress may not correlate well with the traditional depression phenotype (47). The absence of behavioral correlates of depression may reflect a different phenotype of depression in BPD with less core motor retardation and withdrawal. In particular, traditional clinical assessment tools may typically lack the resolution to discern these differential phenotypes, compared to the digital behavior metrics used in our study. Interestingly, our results add to a body of work suggesting that high QIDS scores in BPD individuals may not represent the same diagnostic entity of depression as in other diagnostic groups (33, 48). Furthermore, models developed to predict depressed mood in other diagnostic groups have translated poorly to BPD (13). If these results continue to be replicated in other domains, it may be that the mental distress reported by BPD individuals is more suitably conceptualized using a different diagnostic term other than depression, to reflect the different experiences, behavioral phenotypes and treatment outcomes associated with mental distress in BPD (49).

Our findings are especially interesting given that BPD is often defined as a clinical disorder of attachment, interpersonal dysfunction, perceived abandonment and the formation of unstable relationships (7). It is possible that the persistence of social interaction during states of self-reported depression in BPD represents a type of mental distress closely associated with and possibly caused by such factors, which results in patients seeking to reaffirm their social relationships and allay perceived abandonment through persistent communication. Alternatively, it is possible that the chronic influence of interpersonal features of BPD simply over-ride any observable influence of mood on communications metrics. It is also possible that the traditional characterization of BPD as a disorder of interpersonal dysfunction results from the persistent seeking of social interaction during states of mental distress compared to other diagnostic cohorts (such as BD), who are deemed to internalize depressed mood and withdraw from social settings in line with social norms and expectations.

Regarding diagnosis, compared to HC, BD was associated with increased total call frequency, and BPD was associated with increased total and outgoing SMS frequency, even after adjustment for mood symptoms. These effects appear to have been largely driven by trait-impulsivity. This is in keeping with previous work in non-clinical populations which has identified associations between trait impulsivity and self-reported, often problematic mobile phone use in non-clinical populations (50–54). Phone-call variables were significantly associated with the motor component of impulsivity, whereas SMS use tended to be associated with the attentional and non-planning components of impulsivity (Supplementary Table 8). Although previous work has associated general mobile phone use with the urgency component of impulsivity (51), we believe this is the first finding of differential associations between components of impulsivity and phone call and SMS messaging. Self-reported trait impulsivity correlates poorly with laboratory assessments of impulsivity in BD (55) and digital communications may therefore represent a novel, ecologically-valid, objective marker of impulsivity if our findings are replicated in larger samples.

### Limitations

Although phone call and SMS communication was frequent during the study period, it cannot be assumed that this represented a participants' complete engagement with digital communications. Social media and instant messaging applications are increasingly used in the general population and involve communication using both live and recorded written, vocal, photographic and video media. This fast-changing social ecosystem presents opportunities for future research, especially in light of previous work suggesting behaviors including propensity to send photographs may correlate with psychological traits and subjective well-being (56, 57). Social contacts may be sensitive to unique signs of illness relapse in individual participants, and incoming communications metrics beyond the scope of this study may therefore be required to detect change more reliably.

Participants were provided with a mobile phone upon study enrolment, and it is possible that they continued to use other phones during the study period. Equally, it is possible that the study phone was lent to others during the observation period. These are currently unavoidable drawbacks of ecological study designs which require trust that participants follow research instructions. Use of an Android device may also have caused a selection bias in our study population and skewed the digital behavior we observed; this has been discussed in the literature previously (28, 58).

Our results should also be interpreted in the context of the multiple analyses performed. Our study did not include adjustment for multiple testing and our results should therefore be considered to be exploratory in nature (59). Future research may focus on more specific and sophisticated measures of communication to further explore the general associations we have identified. Our results should also be interpreted in the context of our study's relatively small sample size. However, the sample size is comparable to previous analyses reported in the literature (28, 30) and our study included significant longitudinal follow-up, generating an extensive data-set.

Remote self-assessments are different from objective clinical assessments. However, it is impractical to achieve high-frequency longitudinal mood monitoring by clinical interview and the tools used in this study are clinically-validated self-report scales (60). It is possible that at extremes of mood states participants were less likely to engage in mood-monitoring, and mania in particular may not be as well-served by self-monitoring as depression. Likewise, the uneven contribution of data from different participants is an important limitation (Table 1), although the effect of this was mitigated in part by the use of random effects models.

Mobile phone communications have previously been associated with extraversion, agreeableness, openness and self-consciousness in non-clinical populations (27, 57). These traits were beyond the scope of this study and it is possible that they may partially explain differences in digital communications between diagnostic groups. Similarly, the unbalanced gender proportions between groups is a further limitation of our study, although the preponderance of female participants in our BPD sample is representative of the wider clinical population. To investigate the possible effect of gender, models were replicated with gender included as a fixed effect for the BD and HC cohorts (Supplementary Tables 10–12). This did not significantly alter the results, suggesting that gender is not a significant confounding factor for the associations we identify.

### Conclusion

Our study highlights the potential to identify novel digital biomarkers of mood and diagnosis and demonstrates how such variables can identify behavioral phenotypes of mental distress specific to diagnostic categories. Future work could extend the associations between mood and a wider range of communications metrics in larger cohorts. The identification of such variables may inform the development of multivariate clinical prediction models for individual patients to support clinical diagnosis, prognosis and passive symptom monitoring.

## Data Availability Statement

The original contributions presented in the study are included in the article/Supplementary Material, further inquiries can be directed to the corresponding author.

## Ethics Statement

This study was reviewed and approved by NRES Committee East of England-Norfolk (13/EE/0288). The participants provided their written informed consent to participate in this study.

## Author Contributions

The AMoSS study was conceived and designed by KS and GMG. The Android application was developed by NP. Data was collected by KS and AB. The analyses presented here were conceived, designed and performed by GG. The manuscript was written by GG. All authors contributed to manuscript revision and read and approved the submitted version.

## Conflict of Interest

GMG is a NIHR Emeritus Senior Investigator, holds shares in P1vital and P1Vital products and has served as consultant, advisor or CME speaker in the last 3 years for Compass pathways, Evapharm, Janssen, Lundbeck, Medscape, P1Vital, Sage, Servier. KS is supported by the NIHR Oxford Health Biomedical Research Centre. AB has received salaries from P1vital Ltd. NP holds shares in SmartCare Analytics Ltd. The views expressed are those of the author(s) and not necessarily those of the NHS, the NIHR or the Department of Health. The remaining authors declare that the research was conducted in the absence of any commercial or financial relationships that could be construed as a potential conflict of interest.
