# Supplementary material for: Digital Communication Biomarkers of Mood and Diagnosis in Borderline Personality Disorder, Bipolar Disorder, and Healthy Control Populations
Source: Front Psychiatry. 2021 Apr 8;12:610457. doi: 10.3389/fpsyt.2021.610457 (PMC8060643; doi:10.3389/fpsyt.2021.610457)
Supplement: Supplementary file 1 [file Data_Sheet_1.docx]

Supplementary Material

# Supplementary Tables

## Supplementary Table 1: Communications data by mood symptoms; secondary variables

a. Transdiagnostic model adjusted by age only. All significant results remained significant when age removed from model. b. Adjusted model adjusted for both age and diagnosis. c. Analyses were performed separately for each variable in univariate analyses and with QIDS & ASRM variables together in multivariate analyses. Results remained significant when univariate analysis performed, multivariate analyses results presented here.

|  | Transdiagnostic model^a^ | | | Adjusted by diagnosis^b^ | | |
| --- | --- | --- | --- | --- | --- | --- |
|  | ***Coefficient*** | ***S.E.*** | ***p value*** | ***Coefficient*** | ***S.E.*** | ***p value*** |
| PHONE-CALL |  |  |  |  |  |  |
| Cumulative total call duration^c^ |  |  |  |  |  |  |
| Depressive symptoms (QIDS) | 15.569 | 16.250 | 0.338 | 11.055 | 16.896 | 0.513 |
| Manic symptoms (ASRM) | 70.913 | 24.325 | 0.004 ** | 67.472 | 24.493 | 0.006 ** |
| Cumulative incoming call duration |  |  |  |  |  |  |
| Depressive symptoms (QIDS) | 24.417 | 8.936 | 0.006 ** | 23.185 | 9.528 | 0.015 * |
| Manic symptoms (ASRM) | 33.496 | 13.694 | 0.015 * | 32.972 | 13.853 | 0.017 * |
| Cumulative outgoing call duration |  |  |  |  |  |  |
| Depressive symptoms (QIDS) | -8.635 | 10.920 | 0.429 | -12.550 | 11.347 | 0.269 |
| Manic symptoms (ASRM) | 37.696 | 16.349 | 0.021 * | 34.503 | 16.453 | 0.036 * |
| SMS |  |  |  |  |  |  |
| Cumulative total SMS length |  |  |  |  |  |  |
| Depressive symptoms (QIDS) | 6.09 | 22.52 | 0.787 | -0.293 | 22.706 | 0.990 |
| Manic symptoms (ASRM) | 58.81 | 32.79 | 0.073 | 55.424 | 32.833 | 0.092 |
| Cumulative outgoing SMS length |  |  |  |  |  |  |
| Depressive symptoms (QIDS) | -0.992 | 12.720 | 0.938 | -3.935 | 12.801 | 0.759 |
| Manic symptoms (ASRM) | 18.654 | 18.490 | 0.313 | 17.081 | 18.508 | 0.356 |

## Supplementary Table 2: Communications data by mood state

a. Transdiagnostic model adjusted by age only. All significant results remained significant when age removed from model. b. Adjusted model adjusted for both age and diagnosis.

|  | Transdiagnostic model^a^ | | | Adjusted by diagnosis^b^ | | |
| --- | --- | --- | --- | --- | --- | --- |
|  | ***Coefficient*** | ***S.E.*** | ***p value*** | ***Coefficient*** | ***S.E.*** | ***p value*** |
| PHONE-CALL |  |  |  |  |  |  |
| Total call frequency |  |  |  |  |  |  |
| Depression vs euthymia | 0.593 | 0.769 | 0.441 | 0.430 | 0.784 | 0.584 |
| Mania vs euthymia | 5.162 | 1.121 | 4.44e-06 *** | 5.002 | 1.124 | 9.18e-06 *** |
| Mixed vs euthymia | 2.179 | 1.752 | 0.214 | 1.963 | 1.761 | 0.265 |
| Outgoing call frequency |  |  |  |  |  |  |
| Depression vs euthymia | 0.220 | 0.517 | 0.670 | 0.114 | 0.528 | 0.829 |
| Mania vs euthymia | 3.409 | 0.754 | 6.62e-06 *** | 3.307 | 0.756 | 1.31e-05 *** |
| Mixed vs euthymia | 1.829 | 1.179 | 0.121 | 1.691 | 1.185 | 0.154 |
| Mean total call duration |  |  |  |  |  |  |
| Depression vs euthymia | 31.45 | 16.19 | 0.052 | 30.05 | 16.98 | 0.077 |
| Mania vs euthymia | 33.66 | 24.07 | 0.162 | 31.47 | 24.27 | 0.195 |
| Mixed vs euthymia | 28.09 | 38.07 | 0.461 | 25.84 | 38.50 | 0.502 |
| Mean incoming call duration |  |  |  |  |  |  |
| Depression vs euthymia | 28.283 | 19.961 | 0.157 | 24.057 | 21.521 | 0.264 |
| Mania vs euthymia | 46.802 | 30.058 | 0.120 | 45.753 | 30.455 | 0.133 |
| Mixed vs euthymia | 158.847 | 48.009 | 0.001 *** | 155.098 | 48.754 | 0.002 ** |
| Mean outgoing call duration |  |  |  |  |  |  |
| Depression vs euthymia | -6.769 | 19.655 | 0.731 | -12.825 | 20.690 | 0.535 |
| Mania vs euthymia | 18.362 | 29.417 | 0.533 | 13.558 | 29.675 | 0.648 |
| Mixed vs euthymia | -47.857 | 45.976 | 0.298 | -55.461 | 46.510 | 0.233 |
| SMS |  |  |  |  |  |  |
| Total SMS frequency |  |  |  |  |  |  |
| Depression vs euthymia | -3.788 | 3.027 | 0.211 | -4.399 | 3.038 | 0.148 |
| Mania vs euthymia | 2.840 | 4.360 | 0.515 | 2.521 | 4.361 | 0.563 |
| Mixed vs euthymia | 25.236 | 6.817 | 0.0002 *** | 24.650 | 6.822 | 0.0003 *** |
| Outgoing SMS frequency |  |  |  |  |  |  |
| Depression vs euthymia | -2.161 | 1.535 | 0.159 | -2.466 | 1.540 | 0.109 |
| Mania vs euthymia | 1.145 | 2.210 | 0.604 | 0.991 | 2.210 | 0.654 |
| Mixed vs euthymia | 11.393 | 3.455 | 0.001 *** | 11.088 | 3.457 | 0.001 ** |
| Mean total SMS length |  |  |  |  |  |  |
| Depression vs euthymia | -2.879 | 5.139 | 0.576 | -1.695 | 5.575 | 0.761 |
| Mania vs euthymia | -15.593 | 8.036 | 0.053 | -12.988 | 8.141 | 0.111 |
| Mixed vs euthymia | -0.311 | 12.416 | 0.980 | 1.838 | 12.597 | 0.884 |
| Mean outgoing SMS length |  |  |  |  |  |  |
| Depression vs euthymia | 0.113 | 4.572 | 0.980 | -0.944 | 4.780 | 0.843 |
| Mania vs euthymia | -1.883 | 6.835 | 0.783 | -0.991 | 6.891 | 0.886 |
| Mixed vs euthymia | 5.587 | 10.193 | 0.584 | 4.861 | 10.293 | 0.637 |

## Supplementary Table 3: Communications data by mood state; secondary variables

a. Transdiagnostic model adjusted by age only. All significant results remained significant when age removed from model. b. Adjusted model adjusted for both age and diagnosis.

|  | Transdiagnostic model^a^ | | | Adjusted by diagnosis^b^ | | |
| --- | --- | --- | --- | --- | --- | --- |
|  | ***Coefficient*** | ***S.E.*** | ***p value*** | ***Coefficient*** | ***S.E.*** | ***p value*** |
| PHONE-CALL |  |  |  |  |  |  |
| Cumulative total call duration |  |  |  |  |  |  |
| Depression vs euthymia | 48.566 | 182.280 | 0.790 | 11.173 | 185.035 | 0.952 |
| Mania vs euthymia | 1344.037 | 264.552 | 4.24e-07 *** | 1320.867 | 265.205 | 7.08e-07 *** |
| Mixed vs euthymia | 494.383 | 413.809 | 0.232 | 448.839 | 415.417 | 0.280 |
| Cumulative incoming call duration |  |  |  |  |  |  |
| Depression vs euthymia | 217.234 | 102.737 | 0.035 * | 197.595 | 105.420 | 0.061 |
| Mania vs euthymia | 581.578 | 150.386 | 0.0001 *** | 576.869 | 151.073 | 0.0001 *** |
| Mixed vs euthymia | 589.314 | 235.076 | 0.012 * | 568.922 | 236.649 | 0.016 * |
| Cumulative outgoing call duration |  |  |  |  |  |  |
| Depression vs euthymia | -164.555 | 122.569 | 0.180 | -191.394 | 124.406 | 0.124 |
| Mania vs euthymia | 766.794 | 177.902 | 1.74e-05 *** | 745.265 | 178.302 | 3.09e-05 *** |
| Mixed vs euthymia | -99.720 | 278.271 | 0.720 | -133.889 | 279.297 | 0.632 |
| SMS |  |  |  |  |  |  |
| Cumulative total SMS length |  |  |  |  |  |  |
| Depression vs euthymia | -0.339 | 247.549 | 0.999 | -44.193 | 248.295 | 0.859 |
| Mania vs euthymia | 350.079 | 356.358 | 0.326 | 334.021 | 356.442 | 0.349 |
| Mixed vs euthymia | 553.090 | 557.159 | 0.321 | 508.874 | 557.514 | 0.362 |
| Cumulative outgoing SMS length |  |  |  |  |  |  |
| Depression vs euthymia | -62.676 | 139.507 | 0.653 | -82.68 | 139.83 | 0.554 |
| Mania vs euthymia | 17.238 | 200.730 | 0.932 | 10.38 | 200.77 | 0.959 |
| Mixed vs euthymia | 262.669 | 313.802 | 0.403 | 240.73 | 313.96 | 0.443 |

## Supplementary Table 4: Communications preference (phone-call variables standardised to SMS variables) by mood state and manic symptoms

a. Unadjusted model is adjusted by age only. All significant results remained significant when age removed from model. b. Adjusted model adjusted for both age and diagnosis. c. Call frequency standardised to SMS frequency = (call frequency)/(SMS frequency) d. Call duration standardised to SMS length = (call duration)/(SMS length)

|  | Transdiagnostic model^a^ | | | Adjusted by diagnosis^b^ | | |
| --- | --- | --- | --- | --- | --- | --- |
|  | ***Coefficient*** | ***S.E.*** | ***p value*** | ***Coefficient*** | ***S.E.*** | ***p value*** |
| Total call frequency standardised to SMS frequency^c^ |  |  |  |  |  |  |
| Mania vs euthymia | 0.237 | 0.370 | 0.522 | 0.197 | 0.375 | 0.599 |
| Manic symptoms (ASRM) | -0.033 | 0.032 | 0.307 | -0.039 | 0.032 | 0.234 |
| Outgoing call frequency standardised to SMS frequency |  |  |  |  |  |  |
| Mania vs euthymia | 0.144 | 0.311 | 0.643 | 0.113 | 0.316 | 0.722 |
| Manic symptoms (ASRM) | -0.011 | 0.027 | 0.680 | -0.015 | 0.027 | 0.570 |
| Total call duration standardised to SMS length^d^ |  |  |  |  |  |  |
| Mania vs euthymia | 6.789 | 3.733 | 0.069 | 6.267 | 3.889 | 0.107 |
| Manic symptoms (ASRM) | 0.672 | 0.307 | 0.029* | 0.626 | 0.323 | 0.053 |
| Outgoing call duration standardised to SMS length |  |  |  |  |  |  |
| Mania vs euthymia | 5.942 | 2.567 | 0.021* | 5.256 | 2.647 | 0.047 * |
| Manic symptoms (ASRM) | 0.493 | 0.214 | 0.022 * | 0.405 | 0.221 | 0.067 |

## Supplementary Table 5: Communications data by diagnosis, adjusted by mood state

a. Unadjusted model is adjusted by age only. All significant results remained significant when age removed from model. b. Adjusted model adjusted for both age and mood state.

|  | Unadjusted^a^ | | | Adjusted by mood state^b^ | | |
| --- | --- | --- | --- | --- | --- | --- |
|  | ***Coefficient*** | ***S.E.*** | ***p value*** | ***Coefficient*** | ***S.E.*** | ***p value*** |
| PHONE-CALL |  |  |  |  |  |  |
| Total call frequency |  |  |  |  |  |  |
| BD vs HC | 5.912 | 2.791 | 0.040 * | 5.014 | 2.768 | 0.077 |
| BPD vs HC | 3.561 | 2.835 | 0.216 | 3.129 | 2.856 | 0.279 |
| Outgoing call frequency |  |  |  |  |  |  |
| BD vs HC | 3.624 | 1.866 | 0.059 | 3.055 | 1.841 | 0.104 |
| BPD vs HC | 2.185 | 1.896 | 0.256 | 1.992 | 1.900 | 0.300 |
| Mean total call duration |  |  |  |  |  |  |
| BD vs HC | 42.013 | 39.490 | 0.294 | 30.47 | 40.38 | 0.455 |
| BPD vs HC | 37.596 | 40.345 | 0.357 | 15.01 | 42.72 | 0.727 |
| Mean incoming call duration |  |  |  |  |  |  |
| BD vs HC | 20.257 | 40.202 | 0.618 | 2.982 | 40.892 | 0.942 |
| BPD vs HC | 46.534 | 41.316 | 0.268 | 23.497 | 44.325 | 0.599 |
| Mean outgoing call duration |  |  |  |  |  |  |
| BD vs HC | 50.870 | 45.697 | 0.273 | 53.074 | 46.337 | 0.259 |
| BPD vs HC | 38.332 | 46.948 | 0.419 | 49.680 | 49.486 | 0.320 |
| SMS |  |  |  |  |  |  |
| Total SMS frequency |  |  |  |  |  |  |
| BD vs HC | 36.311 | 23.501 | 0.129 | 36.125 | 22.882 | 0.121 |
| BPD vs HC | 54.522 | 23.786 | 0.026 * | 56.107 | 23.238 | 0.019 * |
| Outgoing SMS frequency |  |  |  |  |  |  |
| BD vs HC | 18.164 | 12.176 | 0.142 | 18.216 | 11.967 | 0.134 |
| BPD vs HC | 29.045 | 12.323 | 0.022 * | 30.093 | 12.150 | 0.017 * |
| Mean total SMS length |  |  |  |  |  |  |
| BD vs HC | -21.300 | 9.306 | 0.027 * | -19.049 | 9.600 | 0.053 |
| BPD vs HC | -8.674 | 9.541 | 0.368 | -7.406 | 10.492 | 0.483 |
| Mean outgoing SMS length |  |  |  |  |  |  |
| BD vs HC | -14.918 | 10.937 | 0.179 | -14.732 | 11.133 | 0.192 |
| BPD vs HC | 6.619 | 11.162 | 0.556 | 7.073 | 11.763 | 0.550 |

## Supplementary Table 6: Communications data by diagnosis, adjusted by mood symptoms and state; secondary variables

a. Unadjusted model is adjusted by age only. All significant results remained significant when age removed from model. b. Adjusted model adjusted for both age and mood symptoms (QIDS & ASRM). c. Adjusted model adjusted for both age and mood state.

|  | Unadjusted^a^ | | | Adjusted by mood symptoms^b^ | | | Adjusted by mood state^c^ | | |
| --- | --- | --- | --- | --- | --- | --- | --- | --- | --- |
|  | ***Coefficient*** | ***S.E.*** | ***p value*** | ***Coefficient*** | ***S.E.*** | ***p value*** | ***Coefficient*** | ***S.E.*** | ***p value*** |
| PHONE-CALL |  |  |  |  |  |  |  |  |  |
| Cumulative total call duration |  |  |  |  |  |  |  |  |  |
| BD vs HC | 1037.440 | 757.148 | 0.178 | 854.235 | 764.029 | 0.269 | 824.973 | 761.675 | 0.285 |
| BPD vs HC | 983.306 | 768.413 | 0.207 | 784.740 | 798.516 | 0.330 | 943.277 | 781.727 | 0.234 |
| Cumulative incoming call duration |  |  |  |  |  |  |  |  |  |
| BD vs HC | 186.912 | 337.452 | 0.583 | 12.061 | 341.877 | 0.972 | 40.803 | 338.964 | 0.905 |
| BPD vs HC | 456.855 | 343.197 | 0.190 | 136.638 | 364.143 | 0.709 | 289.301 | 351.237 | 0.414 |
| Cumulative outgoing call duration |  |  |  |  |  |  |  |  |  |
| BD vs HC | 862.431 | 502.813 | 0.094 | 856.200 | 503.243 | 0.096 | 796.318 | 504.633 | 0.122 |
| BPD vs HC | 533.477 | 510.337 | 0.302 | 658.718 | 526.641 | 0.217 | 664.672 | 518.152 | 0.206 |
| SMS |  |  |  |  |  |  |  |  |  |
| Cumulative total SMS length |  |  |  |  |  |  |  |  |  |
| BD vs HC | 1830.793 | 2104.469 | 0.388 | 1731.851 | 2103.075 | 0.414 | 1777.481 | 2098.142 | 0.401 |
| BPD vs HC | 4931.968 | 2129.676 | 0.025 * | 4882.490 | 2143.277 | 0.027 * | 4930.393 | 2128.800 | 0.025 * |
| Cumulative outgoing SMS length |  |  |  |  |  |  |  |  |  |
| BD vs HC | 930.19 | 1368.21 | 0.500 | 919.116 | 1371.352 | 0.506 | 939.76 | 1368.02 | 0.495 |
| BPD vs HC | 2927.61 | 1384.30 | 0.039 * | 2959.830 | 1394.777 | 0.039 * | 2969.82 | 1386.78 | 0.037 * |

## Supplementary Table 7: Communications variables by diagnosis, adjusted for trait-impulsivity

a. Unadjusted model is adjusted by age only. All significant results remained significant when age removed from model. b. Adjusted model adjusted for both age and trait-impulsivity (BIS).

|  | Unadjusted^a^ | | | Adjusted by trait-impulsivity^b^ | | |
| --- | --- | --- | --- | --- | --- | --- |
|  | ***Coefficient*** | ***S.E.*** | ***p value*** | ***Coefficient*** | ***S.E.*** | ***p value*** |
| Total call frequency |  |  |  |  |  |  |
| BD vs HC | 5.912 | 2.791 | 0.040 * | 2.903 | 3.004 | 0.340 |
| BPD vs HC | 3.561 | 2.835 | 0.216 | 1.533 | 3.128 | 0.627 |
| Total SMS frequency |  |  |  |  |  |  |
| BD vs HC | 36.311 | 23.501 | 0.129 | 14.446 | 25.875 | 0.579 |
| BPD vs HC | 54.522 | 23.786 | 0.026 * | 26.586 | 27.007 | 0.330 |
| Outgoing SMS frequency |  |  |  |  |  |  |
| BD vs HC | 18.164 | 12.176 | 0.142 | 7.246 | 13.481 | 0.593 |
| BPD vs HC | 29.045 | 12.323 | 0.022 * | 15.393 | 14.072 | 0.280 |
| Mean total SMS length |  |  |  |  |  |  |
| BD vs HC | -21.300 | 9.306 | 0.027 * | -20.093 | 10.568 | 0.063 |
| BPD vs HC | -8.674 | 9.541 | 0.368 | -6.501 | 10.909 | 0.554 |
| Cumulative total SMS length |  |  |  |  |  |  |
| BD vs HC | 1830.793 | 2104.469 | 0.388 | 47.32 | 2350.61 | 0.984 |
| BPD vs HC | 4931.968 | 2129.676 | 0.025 * | 2824.02 | 2453.95 | 0.256 |
| Cumulative outgoing SMS length |  |  |  |  |  |  |
| BD vs HC | 930.19 | 1368.21 | 0.500 | 98.73 | 1553.78 | 0.950 |
| BPD vs HC | 2927.61 | 1384.30 | 0.039 * | 1945.76 | 1622.45 | 0.236 |

## Supplementary Table 8: Communications variables by transdiagnostic trait-impulsivity

a. Model is adjusted by age. All significant results remained significant when age removed from model. Separate models were performed for each sub-category of BIS-11 (attentional, non-planning, motor)

|  | Adjusted by age^a^ | | |
| --- | --- | --- | --- |
|  | ***Coefficient*** | ***S.E.*** | ***p value*** |
| Total call frequency |  |  |  |
| BIS-11 | 0.202 | 0.100 | 0.050 * |
| Attentional | 0.254 | 0.277 | 0.364 |
| Non-planning | 0.379 | 0.223 | 0.096 |
| Motor | 0.559 | 0.249 | 0.030 * |
| Total SMS frequency |  |  |  |
| BIS-11 | 2.716 | 0.868 | 0.003 ** |
| Attentional | 7.322 | 2.319 | 0.003 ** |
| Non-planning | 5.391 | 1.918 | 0.007 ** |
| Motor | 3.492 | 2.348 | 0.143 |
| Outgoing SMS frequency |  |  |  |
| BIS-11 | 1.363 | 0.454 | 0.004 ** |
| Attentional | 3.548 | 1.219 | 0.005 ** |
| Non-planning | 2.609 | 1.005 | 0.012 * |
| Motor | 1.994 | 1.213 | 0.107 |
| Mean total SMS length |  |  |  |
| BIS-11 | -0.624 | 0.358 | 0.087 |
| Attentional | -1.102 | 0.974 | 0.263 |
| Non-planning | -1.595 | 0.796 | 0.051 |
| Motor | -0.933 | 0.905 | 0.308 |
| Cumulative total SMS length |  |  |  |
| BIS-11 | 208.044 | 79.666 | 0.012 * |
| Attentional | 482.22 | 216.61 | 0.031 * |
| Non-planning | 376.34 | 176.37 | 0.038 * |
| Motor | 387.65 | 207.50 | 0.067 |
| Cumulative outgoing SMS length |  |  |  |
| BIS-11 | 106.20 | 52.76 | 0.050 * |
| Attentional | 229.93 | 143.08 | 0.114 |
| Non-planning | 171.769 | 116.288 | 0.146 |
| Motor | 239.594 | 134.480 | 0.081 |

## Supplementary Table 9: Communications data by diagnosis & mood state interaction effects; secondary variables

Data limited to depression & euthymia weeks (n=1380) to avoid rank deficiency. All analyses are adjusted for age. All significant results remained significant when age removed from model. a. In dummy coding, HC group used as reference level, therefore diagnosis x depression represents the moderation effect compared to reference (HC). b. In dummy coding, BPD group used as reference level, therefore diagnosis x depression represents the moderation effect compared to reference (BPD).

|  | Reference: HC group^a^ | | | Reference: BPD group^b^ | | |
| --- | --- | --- | --- | --- | --- | --- |
|  | ***Coefficient*** | ***S.E.*** | ***p value*** | ***Coefficient*** | ***S.E.*** | ***p value*** |
| PHONE-CALL |  |  |  |  |  |  |
| Cumulative total call duration |  |  |  |  |  |  |
| Depression | 333.482 | 298.738 | 0.264 | 392.996 | 293.066 | 0.180 |
| BD vs HC | 1457.430 | 788.883 | 0.071 | - | - | - |
| BPD vs HC | 755.736 | 830.864 | 0.367 | - | - | - |
| BD vs BPD | - | - | - | 701.694 | 872.481 | 0.425 |
| BD x Depression | -1598.620 | 464.923 | 0.001 *** | -1658.134 | 461.222 | 0.0003 *** |
| BPD x Depression | 59.514 | 418.485 | 0.887 | - | - | - |
| Cumulative incoming call duration |  |  |  |  |  |  |
| Depression | 368.649 | 171.192 | 0.031 * | 261.073 | 167.512 | 0.119 |
| BD vs HC | 289.505 | 354.500 | 0.418 | - | - | - |
| BPD vs HC | 280.634 | 380.174 | 0.463 | - | - | - |
| BD vs BPD | - | - | - | 8.871 | 401.307 | 0.982 |
| BD x Depression | -702.303 | 264.361 | 0.008 ** | -594.726 | 261.925 | 0.023 * |
| BPD x Depression | -107.577 | 239.509 | 0.653 | - | - | - |
| Cumulative outgoing call duration |  |  |  |  |  |  |
| Depression | -41.057 | 206.117 | 0.842 | 121.226 | 202.135 | 0.549 |
| BD vs HC | 1165.957 | 524.553 | 0.032 * | - | - | - |
| BPD vs HC | 486.657 | 553.679 | 0.384 | - | - | - |
| BD vs BPD | - | - | - | 679.300 | 581.840 | 0.249 |
| BD x Depression | -875.947 | 320.467 | 0.006 ** | -1038.230 | 317.863 | 0.001 ** |
| BPD x Depression | 162.283 | 288.689 | 0.574 | - | - | - |
| SMS |  |  |  |  |  |  |
| Cumulative total SMS length |  |  |  |  |  |  |
| Depression | 110.86 | 402.10 | 0.783 | 396.92 | 396.16 | 0.317 |
| BD vs HC | 1979.85 | 2560.52 | 0.443 | - | - | - |
| BPD vs HC | 5224.99 | 2636.12 | 0.053 | - | - | - |
| BD vs BPD | - | - | - | -3245.14 | 2739.04 | 0.242 |
| BD x Depression | -1463.73 | 632.69 | 0.021 * | -1749.79 | 628.92 | 0.005 ** |
| BPD x Depression | 286.06 | 564.47 | 0.612 | - | - | - |
| Cumulative outgoing SMS length |  |  |  |  |  |  |
| Depression | -8.637 | 227.812 | 0.970 | 16.50 | 224.47 | 0.941 |
| BD vs HC | 1034.225 | 1419.326 | 0.499 | - | - | - |
| BPD vs HC | 3242.493 | 1563.565 | 0.043 * | - | - | - |
| BD vs BPD | - | - | - | -2208.27 | 1624.20 | 0.180 |
| BD x Depression | -578.749 | 358.535 | 0.107 | -603.89 | 356.41 | 0.090 |
| BPD x Depression | 25.139 | 319.821 | 0.937 | - | - | - |

## Supplementary Table 10: Communications data by diagnosis (adjusted by gender)

Data limited to BD and HC cohorts only, to adjust significant results reported in Tables 4 & 5 by gender. a. Unadjusted model is adjusted by age and gender. b. Adjusted model is adjusted for age, gender and mood symptoms (QIDS & ASRM). Data limited to BD and HC cohorts only

|  | Unadjusted^a^ | | | Adjusted by mood symptoms^b^ | | |
| --- | --- | --- | --- | --- | --- | --- |
|  | ***Coefficient*** | ***S.E.*** | ***p value*** | ***Coefficient*** | ***S.E.*** | ***p value*** |
| Total call frequency |  |  |  |  |  |  |
| BD vs HC | 6.378 | 2.096 | 0.005 ** | 6.746 | 2.159 | 0.004 ** |
| Mean total SMS length |  |  |  |  |  |  |
| BD vs HC | -20.286 | 10.115 | 0.053 | -15.250 | 10.379 | 0.150 |

## Supplementary Table 11: Communications data by diagnosis & mood state interaction effects (adjusted by gender)

Data limited to BD and HC cohorts only, to adjust results reported in Table 6 by gender. All analyses are adjusted for age and gender. Data limited to depression & euthymia weeks to avoid rank deficiency (n=1,008). In dummy coding, HC group used as reference level, therefore diagnosis x depression represents the moderation effect compared to reference (HC).

|  | Reference: HC group^a^ | | |
| --- | --- | --- | --- |
|  | ***Coefficient*** | ***S.E.*** | ***p value*** |
| Total call frequency |  |  |  |
| BD vs HC | 7.613 | 2.125 | 0.001 * |
| BD x Depression | -3.946 | 1.970 | 0.045 * |
| Outgoing call frequency |  |  |  |
| BD vs HC | 4.499 | 1.452 | 0.004 ** |
| BD x Depression | -1.292 | 1.344 | 0.337 |
| Mean total call duration |  |  |  |
| BD vs HC | 56.499 | 49.539 | 0.263 |
| BD x Depression | -133.894 | 43.765 | 0.002 ** |
| Mean incoming call duration |  |  |  |
| BD vs HC | 18.231 | 51.934 | 0.728 |
| BD x Depression | -126.490 | 52.183 | 0.016 * |
| Mean outgoing call duration |  |  |  |
| BD vs HC | 86.788 | 54.915 | 0.125 |
| BD x Depression | -125.620 | 53.745 | 0.020 * |

## Supplementary Table 12: SMS data by diagnosis & mood state interaction effects (adjusted by gender)

Data limited to BD and HC cohorts only, to adjust results reported in Table 7 by gender. All analyses are adjusted for age and gender. Data limited to depression & euthymia weeks to avoid rank deficiency (n=1,008). In dummy coding, HC group used as reference level, therefore diagnosis x depression represents the moderation effect compared to reference (HC).

|  | Reference: HC group^a^ | | |
| --- | --- | --- | --- |
|  | ***Coefficient*** | ***S.E.*** | ***p value*** |
| Total SMS frequency |  |  |  |
| BD vs HC | 43.528 | 16.039 | 0.011 * |
| BD x Depression | -26.070 | 7.421 | <0.001 *** |
| Outgoing SMS frequency |  |  |  |
| BD vs HC | 21.546 | 7.903 | 0.010 * |
| BD x Depression | -11.126 | 3.670 | 0.002 *** |
| Mean total SMS length |  |  |  |
| BD vs HC | -15.161 | 10.637 | 0.162 |
| BD x Depression | -19.481 | 15.633 | 0.213 |
| Mean outgoing SMS length |  |  |  |
| BD vs HC | -12.626 | 12.072 | 0.304 |
| BD x Depression | -4.300 | 12.480 | 0.731 |

# Supplementary Figures

## Supplementary Figure 1: Relationship between depressive symptoms and phone-call variables, by diagnostic group

A scatter plot displaying the relationship between depressive symptoms and cumulative total call duration. Each point corresponds to a participants’ depressive symptoms (measured by QIDS) and the stated phone-call variable in the six days preceding, and day of, a completed mood assessment. Colour coding corresponds to diagnosis. Trendline coefficients are taken from linear mixed-effects regression models adjusted for age. Phone-call variables are; A. Total call frequency (no. of phone calls in the six days preceding, and day of, a completed mood assessment) B. Outgoing call frequency (no. of phone calls in the six days preceding, and day of, a completed mood assessment) C. Mean total call duration (seconds per call) D. Mean outgoing call duration (seconds per call) E. Mean incoming call duration (seconds per call).

## Supplementary Figure 2: Relationship between depressive symptoms and SMS variables, by diagnostic group

A scatter plot displaying the relationship between depressive symptoms and cumulative total call duration. Each point corresponds to a participants’ depressive symptoms (measured by QIDS) and the stated SMS variable in the six days preceding, and day of, a completed mood assessment. Colour coding corresponds to diagnosis. Trendline coefficients are taken from linear mixed-effects regression models adjusted for age. SMS variables are; A. Total SMS frequency (no. of SMS in the six days preceding, and day of, a completed mood assessment) B. Outgoing SMS frequency (no. of SMS in the six days preceding, and day of, a completed mood assessment) C. Mean total SMS length (characters per SMS) D. Mean outgoing SMS length (characters per SMS) E. Cumulative total SMS length (total characters in the six days preceding, and day of, a completed mood assessment) & F. Cumulative outgoing SMS length (total characters in the six days preceding, and day of, a completed mood assessment).
